# Clinicopathological response to neoadjuvant therapies and pathological complete response as a biomarker of survival in human epidermal growth factor receptor-2 enriched breast cancer – A retrospective cohort study

**DOI:** 10.1016/j.breast.2021.06.005

**Published:** 2021-06-18

**Authors:** Matthew G. Davey, Eoin Kerin, C. O'Flaherty, Elizabeth Maher, Vinitha Richard, Peter McAnena, Ray P. McLaughlin, Karl J. Sweeney, Michael K. Barry, Carmel M. Malone, William Wyns, Osama Soliman, Nicola Miller, Maccon M. Keane, Aoife J. Lowery, Michael J. Kerin

**Affiliations:** a The Lambe Institute for Translational Research, National University of Ireland, Galway, Ireland; b Department of Surgery, Galway University Hospitals, Galway, Ireland; c Department of Cardiology, Galway University Hospitals, Galway, Ireland; d Department of Medical Oncology, Galway University Hospitals, Galway, Ireland

**Keywords:** Breast cancer, HER2 gene, Pathological complete response, Personalised medicine, Precision medicine

## Abstract

**Background:**

Human epidermal growth factor receptor-2 (HER2) is overexpressed in 20–25% of breast cancers. Complete eradication of disease following neoadjuvant therapies and chemotherapy has been referred to as pathological complete response (pCR).

**Aims:**

To determine clinicopathological predictors of pCR to neoadjuvant therapies and to evaluate pCR as a surrogate to enhanced survival.

**Methods:**

Consecutive female patients with HER2 positive (HER+) breast cancer managed surgically in a single institution between 2005 and 2015 were included. Descriptive statistics and binary logistic regression were used to determine predictors of pCR. Appraisal of pCR as a predictor of survival was performed using Kaplan-Meier curves and Cox regression analysis.

**Results:**

451 patients were included with a mean age of 56.6 ± 13.4 years (range 23–95). Disease-free (DFS) and overall survival (OS) was 82.3% (371/451) and 82.6% (376/451) respectively with a median follow-up of 108.0 months (range 3–184.0). 118 were treated in the neoadjuvant setting (26.2%): tumour size <50 mm (Odds Ratio (OR): 12.156, *P* = 0.023) and progesterone receptor negativity (OR: 2.762, *P* = 0.008) independently predicted breast pCR, while ductal carcinoma (OR: 3.203, *P* = 0.030) and grade 3 disease (OR: 2.788, *P* = 0.018) predicted axillary pCR. Both breast and axillary pCR predicted enhanced DFS (Hazard Ratio (HR): 0.470 & HR: 0.449) and OS (HR: 0.383 & HR: 0.307). Axillary pCR independently predicted improved OS (HR: 0.326).

**Conclusion:**

pCR is sensitive biomarker and surrogate to survival outcomes in HER2+ breast cancer. Patients likely to achieve pCR may be predicted from traditional clinicopathological characteristics and molecular parameters.

## Highlights

• Clinicopathological data predict patients who achieve pCR in HER2+ breast cancer.
• pCR is a sensitive biomarker of survival in HER2 positive breast cancer.
• Patients achieving pCR have reduced risk of breast cancer recurrence and mortality.
• pCR can aid patient prognostication in the era of personalised medicine.

## 1 Introduction

Breast cancer is the most common cancer in women with an incidence of 12.4% in the western world [1]. In recent years, breast cancer has become divided into four distinct intrinsic biological subtypes with diverse clinical characteristics, individual therapeutic options and varying prognosis [2]. Human epidermal growth factor receptor-2 (HER2) tends to be overexpressed in 20–25% of breast carcinoma [3], and amplification of the gene has been proven to play a crucial role in tumour growth and progression [4]. Traditionally, HER2 positive (HER2+) breast cancers were treated with conventional cytotoxic chemotherapy and carried moderate survival outcomes [5], however the advent of Trastuzumab has revolutionised modern oncological practice for this disease, with outcomes comparable to even the most favourable of luminal breast cancers [6].

Oncological practice has progressed in recent years to recognise the inherent value of treating patients with chemotherapy in the neoadjuvant setting, with advantages such as tumour downstaging and increased breast conserving surgery proving beneficial to patients hoping to avoid mastectomy. Moreover, the neoadjuvant prescription of systemic therapies allows for the generation of in-vivo data in relation to tumour sensitivity, which has been illustrated to carry prognostic significance for disease recurrence and survival [7,8]. Pathological complete response (pCR), which involves complete eradication of cancer tissue from the breast and/or axilla, following neoadjuvant chemotherapies has been proposed as a surrogate endpoint for the prediction of long-term clinical outcomes in clinical trials, such as disease-free (DFS) and overall survival (OS) [9,10]. The perceived benefit of achieving pCR within the current oncological paradigm seems clear, leading to multicentre, prospective trials (such the NeoALLTO and NeoSPHERE) including the biomarker as their primary analytical endpoint [10,11]. In a meta-analysis, Broglio et al. demonstrated that patients with HER2+ breast cancer who achieved pCR to neoadjuvant treatment (NAT) had enhanced survival outcomes when compared to those with residual disease [12]. Such results prove promising, moreover while pCR rates of up to 70% have been described in HER2+ disease following NAT prescription [13,14]. In clinical practice, deciphering those likely to achieve such responses proves challenging to the oncologist, although recent evidence from Katayama et al. suggests HER2 3+ on immunohistochemistry (IHC) and grade 3 disease accurately predict pCR to anti-HER2 therapies [15]. In spite of these promising data, patient and tumour heterogeneity may limit such conclusions until validated in independent analyses, and the prognostic value of pCR remains uncertain in certain circles. Accordingly, the aims of the current study were to determine clinicopathological and IHC characteristics predictive of pCR to NAT in a retrospective HER2+ breast cancer cohort and to validate the role of pCR as a surrogate to survival.

## 2 Methods

### 2.1 Patient selection

Local ethical approval was obtained from the Galway Clinical Research Ethics Committee. A single centre, retrospective observational cohort study was undertaken. Consecutive patients diagnosed and treated surgically between January 2005 and December 2015 for HER2+ breast cancer in an Irish tertiary referral centre were included. Patients with metastatic (M1) disease at presentation were excluded. Patients were included via the symptomatic referral pathway and *BreastCheck* mammographic screening service, which is available to women aged 50–69 every two years in the Republic of Ireland. Patients included were identified from a prospectively maintained database at the Department of Surgery. Detailed data regarding patient demographic, tumour and pathological information, neoadjuvant and adjuvant treatment regimens, oncological surgical procedures, disease recurrence and survival outcomes were collected using patient medical records. Breast cancer molecular subtype was allocated on the basis of the 12th St. Gallen expert consensus (2013) [2].

### 2.2 Multidisciplinary approach to treatment

Included patients presented for triple assessment to our specialised tertiary referral centre for breast cancer patient management. Clinical breast examinations were performed by the attending consultant breast surgeon, radiological appraisal was conducted by a specialist breast consultant radiologist by mammography and/or ultrasound scanning. Core tissue biopsies were usually performed under image guidance by the radiologist and analysed by an expert consultant breast pathologist. All cases where then discussed at the multidisciplinary team at the tertiary referral centre and definitive treatment regimens for each patient were determined according to standard best practice protocols [16,17]. Tailored treatment strategies incorporated clinical, radiological, pathological, IHC, patient performance status, family history, as well as the patient's own wishes with regard to treatment. Patients returned to the tertiary referral centre for annual mammographic follow-up. Tumour staging was performed in accordance with the American Joint Committee on Cancer (AJCC), version 8 Guidelines [18].

### 2.3 Histopathologic and immunohistochemistry appraisal

Tumour specimens were analysed in accordance with the 2010 American Society of Clinical Oncology/College of American Pathologists (ASCO/CAP) histopathological consensus guidelines for estrogen (ER) and progesterone (PgR) receptor status [19] and reported using the Allred scoring system [20]. HER2 status was determined using IHC, and patients scoring 2+ proceeded for fluorescence in-situ hybridization to confirm HER2 status. Histopathological tumour grade was determined in accordance to the Elston-Ellis modification of the Scarff-Bloom-Richardson grading system (as per the World Health Organisation Classification of Tumours Guidelines) [21]. Appraisal of Ki-67 was performed using MIB1 antibody testing [22]. The 2013 St. Gallen expert panel consensus was used to define molecular subtypes [2]. pCR was defined as ‘absence and total eradication of cancer tissue from the breast and/or axilla following resection’ by the attending consultant histopathologist [23].

### 2.4 Patient follow up

Each patient was followed-up and status recorded through a prospectively maintained institutional database. The median and mean lengths of follow-up were calculated using the reverse Kaplan-Meier method [24]. Data in relation to disease recurrence and survival were obtained from electronic patient medical records. Mortality status and cause of death was confirmed through the Republic of Ireland's National Death Registry. Invasive disease-free survival was defined as ‘freedom from invasive disease recurrence or death’.

### 2.5 Statistical analysis

Clinicopathological, IHC, treatment and clinical outcomes were analysed using descriptive statistics; Fisher's exact (¶), Chi-squared (χ^2^) and one-way analysis of variance (ANOVA, *□*) tests were used as appropriate. All tests of significance were 2-tailed, with *P* < 0.050 indicating statistical significance. Binary logistic regression analyses were performed to determine predictors of those likely to undergo NAT, pCR in the breast and in the axilla respectively, expressed in crude odds ratios (OR) with 95% confidence intervals (CIs). Kaplan-Meier and Log-rank (Mantel-Cox) analyses were performed to determine pCR as a surrogate to improved survival. Cox-regression were used to associate survival with clinicopathologic characteristics expressed as hazard ratios (HR) with 95% CIs. Variables with *P* < 0.050 in univariable analysis were included in the multivariable analysis. Data was analysed using Statistical Package for Social Sciences™ (SPSS™) version 26.0 (International Business Machines Corporation, Armonk, New York).

## 3 Results

### 3.1 Patient demographics and follow up

Four hundred and fifty one consecutive patients met inclusion criteria. The mean age at diagnosis was 56.6 ± 13.4 years (range 23–95 years, median age 55 years). Three hundred and sixty five patients (80.9%) presented via the symptomatic referral pathway, while 86 were assessed via the *BreastCheck* screening program (19.1%). Clinicopathological data and time of treatment are outlined on Table 1. The median follow-up was 108.0 months (range 3–184.0 months). DFS and OS at median follow up was 82.3% (371/451) and 83.6% (376/451) respectively. There was no difference in recurrence rates between the neoadjuvant group (27.1%, 32/118) vs. others (29.7%, 99/333) (*P=*0.638, *¶*), and locoregional recurrence rates were comparable between groups (neoadjuvant group: 5.1%, [6/118] vs. adjuvant group: 5.1% [17/333]) (*P=*1.000, *¶*).

**Table 1:** Clinicopathological and immunohistochemical characteristics and timing of treatment.

Characteristic		Overall group (N = 451)	Neoadjuvant Group (N = 118)	Adjuvant Group (N = 333)	P-value
Age at diagnosis	Mean ± SD (range); median	56.6 ± 13.4, (23–95); 55	51.5 ± 12.0, (23–79); 51	58.4 ± 13.4, (26–95); 57	<0.001∗ □
Symptomatic	N (%)	365 (80.9%)	100 (84.7%)	265 (79.6%)	0.275 ¶
Screening detected		86 (19.1%)	18 (15.2%)	68 (20.4%)	
Invasive cancer	N (%)	439 (97.3%)	118 (100.0%)	321 (96.4%)	0.042∗
Non-invasive cancer		12 (2.7%)	0 (0.0%)	12 (3.6%)	
Grade 1	N (%)	4 (0.9%)	1 (0.8%)	3 (0.9%)	0.121 χ^2^
Grade 2		164 (36.4%)	52 (44.1%)	112 (33.6%)	
Grade 3		258 (57.2%)	62 (52.6%)	196 (58.9%)	
Unknown		25 (5.5%)	3 (2.5%)	22 (6.7%)	
IDC	N (%)	374 (82.9%)	93 (78.8%)	281 (84.4%)	<0.001∗χ^2^
ILC		23 (5.1%)	4 (3.4%)	19 (5.7%)	
Mixed		5 (1.1%)	2 (1.7%)	3 (0.9%)	
Other		21 (4.7%)	2 (1.7%)	19 (5.7%)	
IBC		16 (3.5%)	14 (11.9%)	2 (0.6%)	
Unknown		12 (2.7%)	3 (2.5%)	9 (2.7%)	
T1	N (%)	139 (30.8%)	11 (9.3)	128 (38.4%)	<0.001∗χ^2^
T2		216 (47.9%)	55 (46.6%)	161 (48.3%)	
T3		28 (6.2%)	17 (14.4%)	11 (9.3%)	
T4		36 (8.0%)	17 (14.4%)	9 (7.1%)	
TX		37 (8.2%)	18 (15.3%)	19 (8.4%)	
N0	N (%)	202 (44.8%)	20 (17.9%)	183 (55.0%)	<0.001∗χ^2^
N1		137 (30.4%)	58 (49.2%)	79 (23.7%)	
N2		45 (10.0%)	13 (11.0%)	32 (9.6%)	
N3		20 (4.4%)	3 (2.5%)	17 (5.1%)	
NX		47 (10.4%)	20 (16.9%)	27 (8.1%)	
ER positive	N (%)	278 (61.6%)	66 (55.9%)	211 (63.4%)	0.153 ¶
ER negative		173 (38.4%)	52 (44.1%)	121 (36.3%)	
PgR positive	N (%)	216 (47.9%)	56 (47.6%)	160 (48.0%)	0.915 ¶
PgR negative		235 (52.1%)	62 (52.4%)	173 (52.0%)	
LBBC	N (%)	295 (65.4%)	70 (59.3%)	225 (67.6%)	0.114 ¶
HER2 enriched		156 (34.6%)	48 (40.7%)	108 (32.4%)	

N; number, SD; standard deviation, IDC; invasive ductal carcinoma, ILC; invasive lobular carcinoma, mixed; mixed histopathological subtype, IBC; inflammatory breast cancer, T1; tumour stage 1, N1; nodal stage 1, SLNB; sentinel lymph node biopsy, ALND; axillary lymph node dissection, ER; estrogen receptor, PgR; progesterone receptor, LBBC; luminal b breast cancer, HER2; human epidermal growth factor receptor-2 □ denotes one-way analysis of variance (ANOVA) test.

¶ denotes Fisher's exact test.

χ^2^ denotes Chi-square test.

∗denotes statistical significance.

### 3.2 Treatment characteristics

**Fig. 1:**
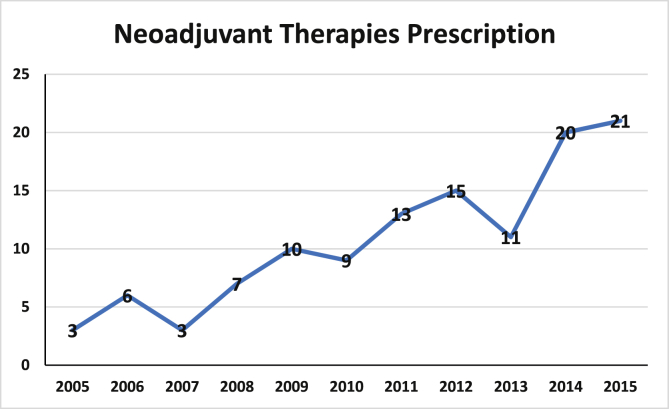
Increase in the prescription of therapies in the neoadjuvant setting in our institution during the course of this study.

Overall, 118 patients were treated in the neoadjuvant setting (26.2%), 55 of whom achieved pCR in the breast and 79 in the axilla (46.6% & 70.0% respectively). Alkylating agents (i.e.: cyclophosphamide, cisplatin, carboplatin, etc.), taxane-based chemotherapy (i.e.: Paclitaxel, Docetaxel, etc.) and Trastuzumab were more likely to be prescribed in the neoadjuvant setting (all *P* < 0.001, *¶*) (Table 2). Patients treated in the neoadjuvant setting were more likely to undergo mastectomy and axillary lymph node dissection (*P* = 0.025 & *P* < 0.001 respectively, *χ*^*2*^). Surgical management and both neoadjuvant and adjuvant systemic treatment characteristics are summarised in Table 2. Fig. 1 illustrates the increase in NAT prescription during the course of this study.

**Table 2:** Treatment characteristics for those treated in the neoadjuvant settings versus other patients.

Procedure	Overall group (N = 451)	Neoadjuvant Group (N = 118)	Other (N = 333)	P-value
Anthracycline-based chemotherapy (i.e.: Doxorubicin, Epirubicin)	38 (8.4%)	6 (5.1%)	32 (9.6%)	0.176 *¶*
Alkylating agents (i.e.: Cyclophosphamide, Carboplatin, Cisplatin)	299 (66.3%)	100 (84.8%)	199 (57.8%)	<0.001∗ *¶*
Taxane-based chemotherapy (i.e.: Docetaxel, Paclitaxel).	304 (67.4%)	107 (99.1%)	197 (59.2%)	<0.001∗ *¶*
Pyrimidine antagonist chemotherapy (i.e.: 5-FU, Capecitabine)	12 (2.7%)	6 (5.1%)	6 (1.5%)	0.148 *¶*
Trastuzamab	334 (74.1%)	104 (88.1%)	230 (69.1%)	<0.001∗ *¶*
Pertuzumab	14 (3.1%)	4 (3.4%)	10 (3.0%)	0.278 *¶*
WLE	285 (63.2%)	65 (55.1%)	220 (66.1%)	0.025∗*χ*^*2*^
Mastectomy	132 (29.3%)	46 (39.0%)	86 (25.8%)	
Missing	34 (7.5%)	7 (5.9%)	27 (8.1%)	
Completion mastectomy	36 (8.0%)	7 (5.9%)	29 (8.7%)	0.496 *χ*^*2*^
Re-excision of margins	21 (4.7%)	4 (3.4%)	17 (5.1%)	
No further breast surgery	394 (87.4)	107 (90.7%)	287 (86.2%)	
SLNB	266 (59.0%)	39 (33.1%)	227 (68.2%)	<0.001∗*χ*^*2*^
ALND	151 (33.5%)	70 (59.3%)	81 (24.3%)	
Missing	34 (7.5%)	9 (7.6%)	25 (7.5%)	
CALND	39 (8.7%)	6 (5.1%)	33 (9.9%)	0.121 *χ*^*2*^
No further axillary surgery	374 (82.9%)	105 (89.0%)	269 (80.8%)	
Missing	38 (8.4%)	7 (5.9%)	31 (9.3%)	
Adjuvant radiotherapy	297 (65.9%)	96 (81.4%)	201 (60.4%)	<0.001∗ *¶*

N; number, 5-FU; 5-Fluorouracil, WLE: wide local excision, SLNB; sentinel lymph node biopsy, ALND; axillary lymph node dissection, CALND; completion axillary lymph node dissection.

¶ denotes Fisher's exact test.

χ^2^ denotes Chi-square test.

∗denotes statistical significance.

### 3.3 Predictors of patients likely to undergo neoadjuvant therapy

Increased tumour and nodal stage were both associated with receiving treatment in the neoadjuvant setting (both *P* < 0.001 *χ*^*2*^) (Table 1). Using univariable analysis, age less than 65 years at the time of diagnosis (Age<65) (OR: 2.751, 95% CI: 1.498–5.054, *P* = 0.001) and tumour size greater than 50 mm (Size >50 mm) (OR: 7.572, 95% CI: 4.100–13.985, *P* < 0.001) both predicted patients likely to receive NAT (Table 3). Using multivariable analysis, both Age<65 (OR: 4.142, 95% CI: 1.940–8.845, *P* < 0.001) & Size >50 mm (OR: 10.404, 95% CI: 5.253–20.604, *P* < 0.001) independently predicted those likely to receive NAT (Table 3).

**Table 3:** Univariate and multivariate binary regression analysis to determine clinicopathological and immunohistochemical predictors of those likely to undergo neoadjuvant treatment.

Parameter	OR	95% CI	P-value	OR	95% CI	P-value
	Univariable			Multivariable		
Age <65 years	2.751	1.498–5.054	0.001∗	4.142	1.940–8.845	<0.001∗
Symptomatic	1.426	0.808–2.516	0.221			
IDC	0.647	0.368 - 1.138	0.131			
Tumour >50 mm	7.573	4.100–13.985	<0.001∗	10.404	5.253–20.604	<0.001∗
Grade 3	0.774	0.507–1.180	0.234			
N2/N3	1.111	0.589–2.063	0.739			
ER negative	1.374	0.897–2.105	0.144			
PgR negative	1.005	0.660–1.531	0.982			
HER2 enriched	1.436	0.930–2.217	0.102			

OR; odds ratio, 95% CI; 95% confidence interval, IDC; invasive ductal carcinoma, N2; nodal stage 2, ER; estrogen receptor, PgR; progesterone receptor, HER2; human epidermal growth factor receptor-2 molecular subtype.

∗denotes statistical significance.

### 3.4 Predictors of patients likely to achieve pCR in the breast

Tumour stage (*P* = 0.031∗*χ*^*2*^), ER negativity (ER-) (*P* = 0.009∗*¶*), PgR negativity (PgR-) (*P* = 0.006∗*¶*) and HER2 enriched molecular subtype (HER2) (*P* = 0.009∗*¶*) were all associated with achieving pCR in the breast (Table 4). Using univariable analysis, size <50 mm (OR: 8.167, 95% CI: 1.001–66.815, *P* = 0.049), ER- (OR: 2.760, 95% CI: 1.303–5.845, *P* = 0.008), PgR- (OR: 2.923, 95% CI: 1.375–6.215, *P* = 0.005) and HER2 (OR: 2.747, 95% CI: 1.288–5.859, *P* = 0.009) predicted patients likely to achieve pCR in the breast. Using multivariable analysis, size <50 mm (OR: 12.156, 95% CI: 1.403–105.299, *P* = 0.023) and PgR- (OR: 2.762, 95% CI: 1.278–5.970, *P* = 0.008) independently predicted achieving pCR in the breast (Table 5).

**Table 4:** Clinicopathological and immunohistochemical characteristics and their relationship with achieving pathological complete response in the breast and axilla.

Characteristic		Neoadjuvant Group (N = 118)	pCR Breast (N = 55)	RD Breast (N = 63)	pCR Axilla (N = 79)	RD Axilla (N = 39)	P-value
Age at diagnosis	Mean ± SD (range); median	51.5 ± 12.0, (23–79); 51	50.7 ± 12.3 (23–77), 50	52.1 ± 11.8 (23–79), 51	50.2 ± 11.7 (23–79), 50	53.4 ± 13.4 (23–79), 51	0.205 *□*
Symptomatic	N (%)	100 (84.7%)	48 (87.3%)	52 (82.5%)	67 (84.8%)	33 (84.6%)	0.612, 0.573
Screening		18 (15.2%)	7 (12.7%)	11 (17.5%)	12 (15.2%)	6 (15.4%)	(Both *¶*)
Grade 1	N (%)	1 (0.8%)	0 (0.0%)	1 (1.6%)	1 (1.3%)	0 (0.0%)	0.709, 0.064(Both *χ*^*2*^)
Grade 2		52 (44.1%)	24 (43.6%)	28 (44.4%)	29 (36.7%)	23 (59.0%)	
Grade 3		62 (52.6%)	29 (52.7%)	33 (52.4%)	48 (60.7%)	14 (35.9%)	
Unknown		3 (2.5%)	2 (3.6%)	1 (1.6%)	1 (1.3%)	2 (5.1%)	
IDC	N (%)	93 (78.8%)	41 (74.6%)	52 (82.5%)	68 (86.1%)	25 (64.1%)	0.226, 0.099(Both *χ*^*2*^)
ILC		4 (3.4%)	1 (1.8%)	3 (4.8%)	1 (1.3%)	3 (7.7%)	
Mixed		2 (1.7%)	1 (1.8%)	1 (1.6%)	1 (1.3%)	1 (2.6%)	
Other		2 (1.7%)	0 (0.0%)	2 (3.2%)	2 (2.6%)	0 (0.0%)	
IBC		14 (11.9%)	10 (18.2%)	4 (6.4%)	5 (6.3%)	9 (23.1%)	
Unknown		3 (2.5%)	2 (3.6%)	1 (1.6%)	2 (2.6%)	1 (2.6%)	
T1	N (%)	11 (9.3)	5 (9.1%)	6 (9.5%)	10 (12.7%)	1 (2.6%)	0.031∗, 0.078 (Both *χ*^*2*^)
T2		55 (46.6%)	20 (36.4%)	35 (55.6%)	37 (46.8%)	18 (46.2%)	
T3		17 (14.4%)	6 (10.9%)	11 (17.5%)	10 (12.7%)	7 (18.0%)	
T4		17 (14.4%)	11 (20.0%)	6 (9.5%)	7 (8.9%)	10 (25.6%)	
TX		18 (15.3%)	13 (23.6%)	5 (7.9%)	15 (19.0%)	3 (7.7%)	
N0	N (%)	20 (17.9%)	8 (14.6%)	12 (19.1%)	20 (25.3%)	0 (0.0%)	0.328, <0.001∗ (Both *χ*^*2*^)
N1		59 (49.2%)	30 (54.5%)	29 (46.0%)	32 (40.5%)	27 (69.2%)	
N2		13 (11.0%)	4 (7.3%)	9 (14.3%)	4 (5.1%)	9 (23.1%)	
N3		3 (2.5%)	0 (0.0%)	3 (4.8%)	0 (0.0%)	3 (7.7%)	
NX		23 (20.4%)	13 (23.6%)	10 (15.9%)	23 (29.1%)	0 (0.0%)	
ER positive	N (%)	66 (55.9%)	23 (41.8%)	43 (68.3%)	41 (51.9%)	25 (64.1%)	0.009∗, 0.362 (Both *¶*)
ER negative		52 (44.1%)	32 (58.2%)	20 (31.7%)	38 (48.1%)	14 (35.9%)	
PgR positive	N (%)	56 (47.6%)	18 (32.7%)	38 (60.3%)	37 (46.8%)	19 (48.7%)	0.006∗, 0.547 (Both *¶*)
PgR negative		62 (52.4%)	37 (67.3%)	25 (39.7%)	42 (53.2%)	20 (51.3%)	
LBBC	N (%)	70 (59.3%)	29 (52.7%)	41 (65.1%)	46 (58.2%)	24 (61.5%)	0.009∗, 0.248 (Both *¶*)
HER2 enriched		48 (40.7%)	26 (47.3%)	22 (34.9%)	33 (41.8%)	15 (38.5%)	

N; number, SD; standard deviation, pCR; pathological complete response, RD; residual disease, IDC; invasive ductal carcinoma, ILC; invasive lobular carcinoma, mixed; mixed histopathological subtype, IBC; inflammatory breast cancer, T1; tumour stage 1, N1; nodal stage 1, SLNB; sentinel lymph node biopsy, ALND; axillary lymph node dissection, ER; estrogen receptor, PgR; progesterone receptor, LBBC; luminal b breast cancer, HER2; human epidermal growth factor receptor-2.

□ denotes one-way analysis of variance (ANOVA) test.

¶ denotes Fisher's exact test.

χ^2^ denotes Chi-square test.

**Table 5:** Univariate and multivariate binary regression analysis to determine clinicopathological and immunohistochemical predictors of those likely to achieve pathological complete response in the 118 patients treated with neoadjuvant systemic therapies.

pCR Breast							pCR Axilla					
Parameter	OR	95% CI	P-value	OR	95% CI	P-value	OR	95% CI	p-value	OR	95% CI	P-value
	Univariable			Multivariable			Univariable			Multivariable		
Age <65 years	1.604	0.503–5.111	0.425				0.349	0.112–1.086	0.069			
Symptomatic	1.394	0.500–3.887	0.526				0.586	0.176–1.947	0.383			
IDC	0.629	0.247 - 1.601	0.331				3.531	1.351 - 9.227	0.010∗	3.203	1.116–9.191	0.030∗
Tumour <50 mm	8.167	1.001–66.815	0.049∗	12.156	1.403–105.299	0.023∗	7.309	1.441–37.701	0.016∗			
Grade 3	0.950	0.460–1.962	0.890				2.572	1.177–5.621	0.018∗	2.788	1.189–6.536	0.018∗
N1	1.917	0.805–4.562	0.141				0.536	0.226–1.270	0.157			
ER negative	2.760	1.303–5.845	0.008∗				1.240	0.576–2.666	0.583			
PgR negative	2.923	1.375–6.215	0.005∗	2.762	1.278–5.970	0.008∗	1.030	0.482–2.198	0.940			
HER2 enriched	2.747	1.288–5.859	0.009∗				1.607	0.732–3.527	0.237			

pCR; pathological complete response, OR; *odds ratio,* 95% CI; 95% confidence interval, IDC; *invasive ductal carcinoma*, N1; nodal stage 1, ER; estrogen receptor, PgR; progesterone receptor, HER2; human epidermal growth factor receptor-2 molecular subtype.

∗denotes statistical significance.

### 3.5 Predictors of patients likely to achieve pCR in the axilla

Nodal stage (*P* < 0.001∗*χ*^*2*^) was associated with achieving pCR in the axilla (Table 4). Using univariable analysis, invasive ductal carcinoma (IDC) (OR: 3.531, 95% CI: 1.351–9.227, *P* = 0.010], size <50 mm (OR: 7.309, 95% CI: 1.441–37.701, *P* = 0.016) and grade 3 disease (OR: 2.572, 95% CI: 1.177–5.621, *P* = 0.018) predicted patients likely to achieve pCR in the axilla. Using multivariable analysis, IDC (OR: 3.203, 95% CI: 1.116–9.191, *P* = 0.030) and grade 3 disease (OR: 2.788, 95% CI: 1.189–6.536, *P* = 0.018) independently predicted patients likely to achieve pCR in the axilla.

### 3.6 Predictors of patients likely to downstage following neoadjuvant therapy

Using univariable analysis, size <50 mm (OR: 4.500, 95% CI: 1.174–17.249, *P* = 0.028), ER- (OR: 3.596, 95% CI: 1.398–9.246, *P* = 0.008), PgR- (OR: 3.595, 95% CI: 1.398–9.246, *P* = 0.008) and HER2 (OR: 3.788, 95% CI: 1.410–10.179, *P* = 0.008) predicted those likely to downstage in the breast, while PgR-independently predicted tumour downstaging following multivariable analysis (OR: 3.590, 95% CI: 1.465–8.797, *P* = 0.005) (Table 6).

**Table 6:** Univariate and multivariate binary regression analysis to determine clinicopathological and immunohistochemical predictors of those likely to achieve tumour downstaging in the 118 patients treated with neoadjuvant systemic therapies.

Downstage Breast							Downstage Axilla					
Parameter	OR	95% CI	P-value	OR	95% CI	P-value	OR	95% CI	p-value	OR	95% CI	P-value
	Univariable			Multivariable			Univariable			Multivariable		
Age <65 years	0.624	0.192–2.6032	0.434				0.625	0.154–2.540	0.511			
Symptomatic	0.493	0.132–1.836	0.292				0.837	0.218–3.208	0.795			
IDC	0.350	0.096 - 1.279	0.112				2.333	0.696 - 8.823	0.170			
Tumour <50 mm	4.500	1.174–17.249	0.028∗				0.493	0.096–2.531	0.397			
Grade 3	1.452	0.674–3.531	0.305				1.081	0.459–2.543	0.859			
N1	1.147	0.355–3.703	0.818				7.500	2.334–24.102	0.001∗	7.159	2.149–23.844	0.001∗
ER negative	3.596	1.398–9.246	0.008∗				2.640	1.083–6.435	0.033∗			
PgR negative	3.595	1.477–8.750	0.005∗	3.590	1.465–8.797	0.005∗	2.857	1.135–7.192	0.026∗			
HER2 enriched	3.788	1.410–10.179	0.008∗				3.524	1.432–8.670	0.006∗	3.661	1.362–9.841	0.010∗

OR; odds ratio, 95% CI; 95% confidence interval, IDC; invasive ductal carcinoma, N1; nodal stage 1, ER; estrogen receptor, PgR; progesterone receptor, HER2; human epidermal growth factor receptor-2 molecular subtype.

∗denotes statistical significance.

Using univariable analysis, having N1 disease (OR: 7.500, 95% CI: 2.334–24.102, *P* = 0.001), ER- (OR: 2.640, 95% CI: 1.083–6.435, *P* = 0.033), PgR- (OR: 2.857, 95% CI: 1.135–7.192, *P* = 0.026) and HER2 (OR: 3.524, 95% CI: 1.432–8.670, *P* = 0.006) predicted those likely to downstage in the axilla, while having N1 disease (OR: 7.159, 95% CI: 2.149–23.844, *P* = 0.001) and (OR: 3.661, 95% CI: 1.362–9.841, *P* = 0.010) HER2 independently predicted axilla downstaging following multivariable analysis (Table 6).

### 3.7 pCR as a surrogate to survival

**Fig. 2:**
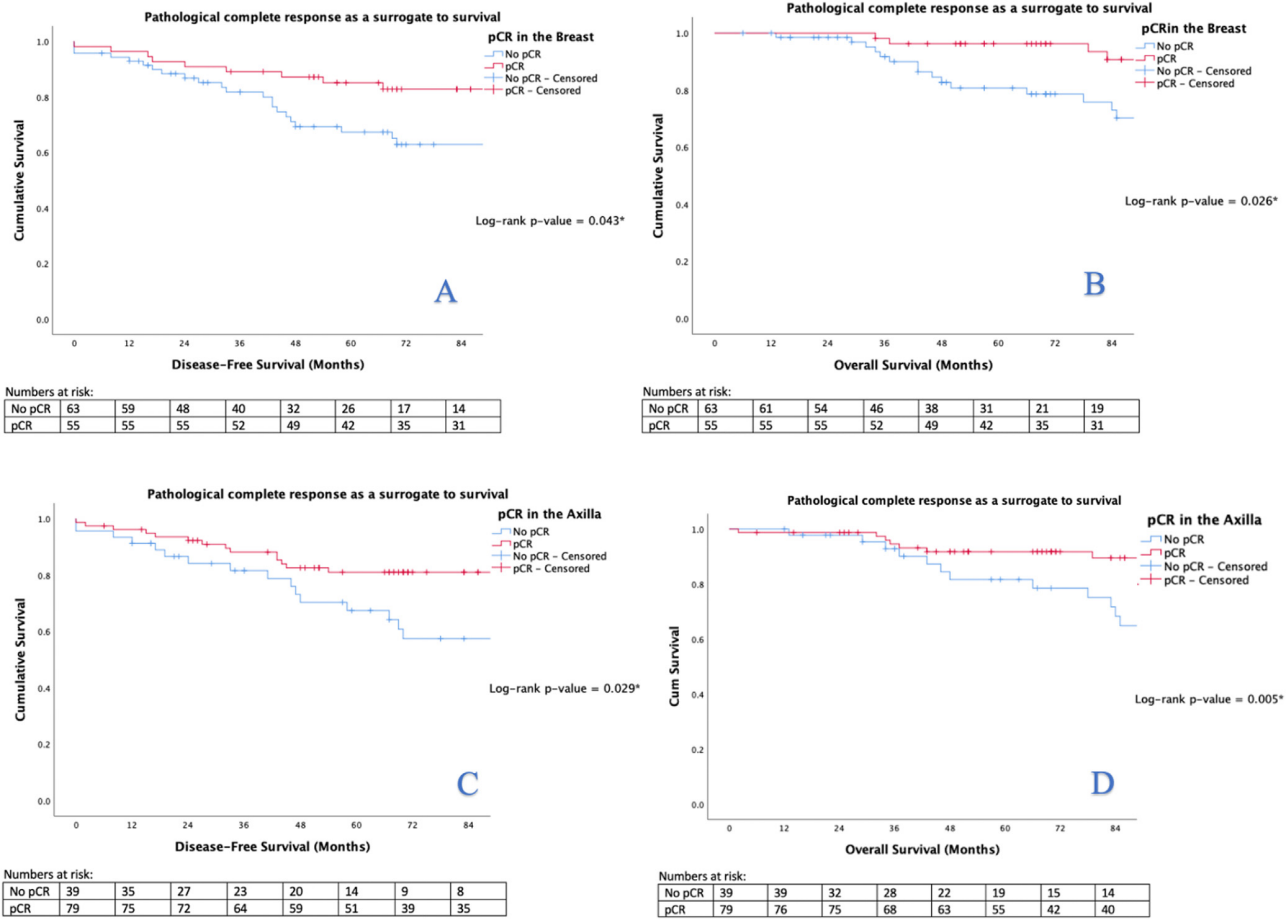
Kaplan-Meier analyses determining the role of pathological complete response in the breast (2A & 2B) and axilla (2C & 2D) as a surrogate biomarker of survival in patients treated with neoadjuvant therapies.

Using univariable analyses, both pCR of the breast (HR: 0.470, 95% CI: 0.222–0.994, *P* = 0.048) and axilla (HR: 0.449, 95% CI: 0.219–0.921, *P* = 0.029) predicted improved DFS when compared to patients with residual disease following NAT. Univariable analyses also demonstrated both pCR of the breast (HR: 0.383, 95% CI: 0.158–0.924, *P* = 0.033) and axilla (HR: 0.307, 95% CI: 0.130–0.729, *P* = 0.007) to predict improved OS. Using multivariable analyses, pCR of the axilla (HR: 0.326, 95% CI: 0.115–0.929, *P* = 0.036) independently predicted improved OS (Table 7). Kaplan-Meier illustrated pCR of both the breast and axilla to be a surrogate marker of improved DFS and OS respectively (all *P* < 0.050, log-rank test) (Fig. 2).

**Table 7:** Univariate and multivariate Cox regression analysis to determine predictors of disease recurrence or death within this series.

DFS							OS					
Parameter	HR	95% CI	P-value	OR	95% CI	P-value	HR	95% CI	p-value	OR	95% CI	P-value
	Univariable			Multivariable			Univariable			Multivariable		
Age >65	2.336	1.485–3.676	<0.001∗				3.343	2.101–5.319	<0.001∗	2.069	1.186–3.611	0.010∗
Symptomatic	19.087	2.656–137.208	0.003∗	13.345	1.836–97.008	0.010∗	14.727	2.044–106.118	0.008∗	8.541	1.163–62.730	0.035∗
IDC	0.577	0.337 - 0.987	0.045∗				0.537	0.307 - 1.937	0.029∗			
Tumour >50 mm	2.001	0.997–4.015	0.051				2.029	1.001–4.109	0.049∗			
Grade 3	1.241	0.786–1.958	0.354				0.995	0.621–1.594	0.982			
N2/N3	2.671	1.603–4.450	<0.001∗	2.570	1.533–4.309	<0.001∗	2.357	1.374–4.042	0.002∗	2.065	1.156–3.688	0.014∗
ER negative	1.319	0.847–2.054	0.221				1.604	1.006–2.557	0.047∗			
PgR negative	1.631	1.030–2.583	0.037∗	1.878	1.125–3.134	0.016∗	1.968	1.192–3.251	0.008∗			
HER2 enriched	1.482	0.949–2.314	0.083				1.796	1.126–2.866	0.014∗			
pCR Breast	0.470	0.222–0.994	0.048∗				0.383	0.158–0.924	0.033∗			
T downstage	0.715	0.318–1.604	0.415				0.904	0.321–2.542	0.848			
pCR Axilla	0.449	0.219–0.921	0.029∗				0.307	0.130–0.729	0.007∗	0.326	0.115–0.929	0.036∗
Axilla downstage	0.622	0.243–1.591	0.322				0.228	0.052–1.006	0.051			

DFS; disease-free survival, OS; overall survival, HR; hazards ratio, 95% CI; 95% confidence interval, IDC; invasive ductal carcinoma, N2; nodal stage 2, ER; estrogen receptor, PgR; progesterone receptor, HER2; human epidermal growth factor receptor-2 molecular subtype, T; tumour, pCR; pathological complete response.

∗denotes statistical significance.

## 4 Discussion

The novel taxonomy of breast cancer into four molecularly distinct subtypes has proven critical in the personalisation of therapeutics [2] and patients with tumours expressing HER2 signalling have proven to be the main beneficiaries following the revolutionary advent of Trastuzumab [25]. In recent times, long-term HER2-blockade has proven crucial in enhancing survival in HER2 enriched cancers [26], transforming the natural history of this disease into one of chronicity, with clinical outcomes comparable to even the most favourable of luminal cancers [27]. Furthermore, following seminal results of the NOAH trial, combined conventional chemotherapy with targeted therapy has become the cornerstone of treatment in neoadjuvant scenarios, with attention turning to pCR in predicting clinical outcomes [28]. The most important finding in the current analysis is data implicating pCR as a surrogate biomarker to survival, with those achieving pCR outperforming those with residual cancer tissue following surgical resection. Moreover, this analysis outlined a 7 fold increase in the prescription of NAT for patients with HER2+ disease in recent times, thus highlighting a real world adoption of this therapeutic strategy.

In this study, patients achieving pCR of the breast or axilla were less than 50% as likely to suffer recurrence or death as their counterparts with residual disease following NAT. Similarly, breast and/or axillary pCR predicted reduced mortality rates of 66% compared to patients with residual disease following NAT. This data in supported by Spring et al. in their recent analysis; these authors advocate for pCR as a predictive biomarker of event free survival in HER2+ breast cancer (HR: 0.32, 95% CI: 0.21–0.47), albeit in the setting of modest pCR rates of 36.4%. pCR has become validated as an endpoint in modern multicentre randomised trials, with both NeoSphere and NeoALTTO trials focusing on pCR resulting from multimodal therapies (including conventional Docetaxel, Trastuzumab and/or Pertuzumab in monotherapy or in combination) in the neoadjuvant setting [10,11,29]. Traditionally, tumour burden has aided clinical prognostication in breast cancer [30], and more recently, residual tumour burden (RTB) has been transformed into a biomarker sensitive for disease recurrence and mortality [31]. The seminal results of the KATHERINE trial illustrate the value of Trastuzumab Emtansine in enhancing clinic-oncological outcomes in those with residual disease following NAT [32], providing promise for those failing to achieve pCR following primary treatment. pCR is the corollary of RTB, and is defined as absence of residual tumour following NAT [23], and therefore it is somewhat unsurprising that pCR is a valuable predictor of enhanced oncological outcome (i.e.: DFS and OS) in the current study. The findings of this study support this; this data, in tandem with those of large multicentre prospective trials [11,29], suggest pCR is a valid analytical endpoint for future prospective oncology trials, particularly with evolving evidence suggesting pCR to be clinically useful in selecting patients for whom a shorter duration of adjuvant anti-HER2 therapy may be sufficient [33].

Within the context of HER2+ disease, there has been variable reported pCR rates following NAT, with rates as high as 70% previously described by Kristle-Whittemore et al. in their analysis of those with HER2 3+ on IHC [13]. Data from this series demonstrates pCR rates of 46.6% and 70.0% in the breast and axilla respectively, consistent with large, prospective analyses [29], despite just four patients being receiving dual anti-HER2 blockade (Table 2). Prediction of pCR rates within HER2+ disease may only be reliable once treatment is indicated in accordance to ‘true’ patient HER2 status, with several factors impacting accurate HER2 measurement: Data from the GeparQuattro study revealed discrepancies in HER2 testing between central and peripheral tumour tissue, with greatest sensitivity to anti-HER2 therapy observed in central tumour tissue (pCR probability rate of 46.8% vs. 20.3% in peripheral tissue) [10]. Moreover, estimating pCR is further complicated by intra-tumour hypoxia within the tumour microenvironment (TME), a known factor to reduce response to therapies [34,35]. ‘True’ HER2 gene expression enhances cellular activity in tumour cells in response to hypoxia, while hypoxia inducible factor 2a (HIF-2a) is thought to regulate oxygenation within TME. Crosstalk between HER2 and HIF-2a seems crucial in regulating tumourgenesis, as well as responses to therapy, with recent data suggesting HIF-2a inhibition is important in improving sensitivity to Trastuzamab [36]. Collectively, these results propose accurate measurement of ‘true’ HER2 expression is crucial in anticipating pCR rates within this disease, while advocating for future directions to be centred around the augmentation of Trastuzumab with novel therapeutic agents, such as HIF-2a inhibitors, in efforts to increase tumour sensitivity and pCR frequencies.

Recent data published from the results of the NeoALTTO study highlight increased pCR rates in those treated with dual anti-HER2 treatment (51.3%) compared to monotherapy with Trastuzumab (29.5%) or Lapatinib (24.7%) alone [29]. In NeoALTTO, pCR significantly predicted enhanced survival [11]. In the current analysis, combined Trastuzumab, alkylating agents and taxane-based chemotherapies (known in combination as ‘TCH’) formed the mainstay neoadjuvant regimen, being prescribed in excess of 85% of cases; this triple agent regimen has now become incorporated into best practice guidelines for early HER2+ breast cancer with up to T2 and/or N2 disease [37]. The inherent value of this combination has been recently validated vs. quadruple therapy following the recent of the NeoCARH multicentre, randomised phase-II trial which established pCR rates of 56.1% following TCH *vs.* 38.5% from quadruple therapy [38], while TCH's favourable safety profile compared to anthracyclines has been highlighted in the results of the BETH trial [39]. In combination, the favourable safety profile and strong affiliation with pCR rates prove combined TCH and surgical resection as the favourable strategy for the vast majority of patients, as has been coherently outlined in this retrospective analysis. Furthermore, the recent work of van der Voort et al. illustrate favourable 3-year outcomes in stage II/III HER2+ disease when omitting anthracycline-based chemotherapy, as well as less drug-induced cardiotoxicities [40].

In the current analysis, it is unsurprising that histopathological and immunohistochemical characteristics such as PgR status, tumour grade and disease burden independently predict breast and axillary pCR, as described in several recent studies [15,[41], [42], [43]]. In their analysis of 2366 breast cancer patients in the Netherlands Cancer Registry treated with NAT, Goorts et al. reported clinical tumour stage to be the most important predictive factor in determining pCR [44]: pCR rates declined steadily from 31% to 17% for those with T1 vs. T4 disease respectively, and pCR rates were substantially higher in patients with T1-T2 disease compared to larger cancers (OR: 3.15, *P* < 0.001). Data from our multivariable model suggests the likelihood of pCR is 12 times more likely in cancers <50 mm, albeit limited to HER2+ disease alone, *vs.* mixed molecular subtyping in Goorts’ analysis. However, patients with HER2+ disease in this study seem dependent upon ER-to achieve breast/axillary downstaging, irrespective of tumour burden, which reciprocates to data published from the Memorial Sloan Kettering Cancer Centre, New York [41]. Given the invaluable role of ER and HER2/neu in dictating the substratification of breast cancer molecular subtypes and providing targeted therapeutic options through endocrine and anti-HER2 agents, this study further highlights the critical importance of these biomarkers in contemporary breast cancer management during the molecular era. Additionally, the authors wish to highlight the recent work from of McNamara et al. illustrating the potential of proteomic tumour analyses in the identification of novel putative biomarkers predictive of pCR in HER2+ early breast cancer [45]. Furthermore, the early results of the seminal PREDIX HER2 trial [NCT: 02568839]) support routine appraisal of tumour infiltrating lymphocytes given their predictive value in determining pCR [46] – such studies illustrate the emphasis on efforts to further personalise care for patients diagnosed with HER2+ breast cancer within the molecular era.

The current analysis is subject to the inherent limitations of being a retrospective cohort study, recruiting patients from a single, tertiary referral centre representing patients from a unique cultural region, on the edge of mainland Europe. Moreover, recent ASCO/CAP guidelines (2021) suggest NAT should be prescribed for the vast majority of patients with HER2+ breast cancer, with exceptions limited to those with T1a/N0 and T1bN0 disease (unless recruited into clinical trial settings) [47]. This implies our data indicates tumour burden as the main predictor of those in receipt of NAT and achieving pCR to brought into question in the evolving oncological paradigm. In spite of the aforementioned limitations, the authors believe this study reflects real world multidisciplinary care and contemporary HER2+ breast cancer patient management in the early 21st century.

In conclusion, results from the current analysis comprehensively support the pre-existing evidence illustrating pCR after NAT is a sensitive biomarker and surrogate to survival in patients being treated for locally advanced HER2+ breast cancer. Patients likely to achieve pCR in both the breast and axilla may be pre-determined using routine clinicopathological and immunohistochemical characteristics such as degree of disease burden, Nottingham tumour grade and steroid hormone receptor status. This study highlights the innate value of NAT in improving survival in patient diagnosed with HER2+ breast cancer, with the natural history of the disease becomes disseminated into one of chronicity with favourable outcomes.

## Sources of funding

M.G.D received funding from the National Breast Cancer Research Institute, Ireland.
